# circFBLIM1 act as a ceRNA to promote hepatocellular cancer progression by sponging miR-346

**DOI:** 10.1186/s13046-018-0838-8

**Published:** 2018-07-27

**Authors:** Ning Bai, Eming Peng, Xingsheng Qiu, Ning Lyu, Zhejia Zhang, Yiming Tao, Xinying Li, Zhiming Wang

**Affiliations:** 1 Department of General Surgery, Xiangya Hospital, Central South University, 87 Xiangya Road, Changsha, Hunan 410008 People’s Republic of China; 2 Department of XIMC Outpatient, Xiangya Hospital, Central South University, Changsha, China; 3 Department of Radiation Oncology, Sun Yat-Sen Memorial Hospital, Sun Yat-sen University, Guangzhou, China; 4 Department of Medical Imaging and Interventional Radiology, Sun Yat-sen University Cancer Center, Guangzhou, China

**Keywords:** Circular RNAs, miR-346, FBLIM1, Competitive endogenous RNAs, Hepatocellular cancer

## Abstract

**Backgroud:**

Accumulating evidences indicate that circular RNAs (circRNAs), a class of non-coding RNAs, play important roles in tumorigenesis. However, the function of circRNAs in hepatocellular cancer (HCC) is largely unknown.

**Methods:**

We performed circRNA microarrays to identify circRNAs that are aberrantly expressed in HCC tissues. Expression levels of a significantly upregulated circRNA, circFBLIM1, was detected by quantitative real-time PCR (qRT-PCR) in HCC cell lines and tissues. Then, we examined the functions of circFBLIM1 in HCC by cell proliferation, apoptosis, invasion and mouse xenograft assay. In addition, luciferase assay and RNA immunoprecipitation (RIP) assay were used to explore the miRNA sponge function of circFBLIM1 in HCC.

**Results:**

Microarray analysis and qRT-PCR verified a circRNA termed circFBLIM1 that was upregulated in HCC tissues and cell lines. Knockdown of circFBLIM1 inhibited proliferation, invasion and promoted apoptosis in HCC. Via luciferase reporter assays, circFBLIM1 and FBLIM1 were observed to directly bind to miR-346. Subsequent experiments showed that circFBLIM1 and FBLIM1 regulated the expression of each other by sponging miR-346.

**Conclusions:**

Taken together, we conclude that circFBLIM1 may function as a competing endogenous RNA (ceRNA) to regulate FBLIM1 expression through sponging miR-346 to exert regulatory functions in HCC. circFBLIM1 may be a diagnostic biomarker and potential target for HCC therapy.

## Background

Liver cancer is a common malignant disease. It is estimated that there were 782,500 new cases and 745,500 deaths of liver cancer during 2012 worldwide [1]. Most primary liver cancer cases are hepatocellular cancer (HCC), which tends to be diagnosed at advanced stage, and the curative treatments are surgical resection or transplantation. Despite the improvement of surgical technique and radio-chemotherapy regimens in the past decades, the prognosis of HCC remains poor [2–4]. Although it has been reported that many genes relate to the carcinogenesis of HCC, the molecular mechanisms remain mostly obscure [5]. Therefore, it urges us to search novel molecular targets to develop more effective therapeutic strategies for HCC.

Circular RNAs (circRNAs) are a class of non-coding RNAs that are widely expressed in mammals [6]. A plenty of circRNAs have been identified, but their potential functions are poorly understood. There are currently few reports describing the role of circRNAs in HCC. Yao Z et al. reported that circZKSCAN1 inhibits HCC cell growth, migration and invasion [7]. Han D et al. found that circRNA MTO1 acts as the sponge of miR-9 to suppress HCC progression [8]. Revealing the role of circRNAs in HCC will be critical for understanding the molecular mechanisms of carcinogenesis and identification of new biomarkers or therapeutic targets for HCC.

miRNAs are endogenous, non-coding, single-stranded 19- to 25-nucleotide RNAs that play vital roles in cancer process [9]. Increasing studies showed that miRNAs are related to HCC carcinogenesis and progression [10, 11]. Therefore, clarifying the function of a specific miRNA to develop a novel therapeutic strategy may shed some light on the effective management of HCC.

It is reported that RNAs can act as competitive endogenous RNAs (ceRNAs) to co-regulate each other by competing for shared miRNAs [12, 13]. circRNAs have also been shown to function as miRNA sponges or ceRNAs [14]. Hansen TB et al. showed that circRNA Sry functions as a miR-138 sponge [15]. Zhong Z et al. found that circRNA-MYLK functions as ceRNA for miR-29a, which could contribute to EMT and the development of bladder cancer [16]. All these findings indicate that circRNAs could function as miRNA sponges to contribute to the regulation of cancers.

In this study, we analyzed the expression profiles of circRNAs in HCC tissues through microarrays. Expression levels of a significantly upregulated circRNA, circFBLIM1, was detected by quantitative real-time PCR (qRT-PCR) in HCC cell lines and tissues. We examined the functions of circFBLIM1 in HCC and found that knockdown of circFBLIM1 could inhibit cell proliferation and invasion, and induce apoptosis. In addition, luciferase assay and RNA immunoprecipitation (RIP) assay showed that circFBLIM1 could bind to miR-346. Furthermore, FBLIM1 was also a direct target of miR-346. Taken together, we conclude that circFBLIM1 may act as a ceRNA to regulate the expression of FBLIM1 by decoying miR-346. circFBLIM1 can be used as a diagnostic biomarker and potential target in HCC therapy.

## Methods

### Clinical specimens

Three pairs of snap-frozen HCC tissues and matched para-carcinoma normal tissues were obtained from Xiangya Hospital, Central South University for circRNA microarray analysis. Subsequently, a total of 50 paired HCC tissues and para-carcinoma normal tissues were used for validation. All the patients were diagnosed by histopathology and did not receive any other treatment prior to operation. This study was approved by the Ethics Committees of Xiangya Hospital, Central South University, and conducted in accordance with the Helsinki Declaration. Informed consents were obtained from all patients.

### Microarray analysis

circRNA microarray analysis were performed using Human CircRNA Array v2.1 (CapitalBio Technology, China). Total RNA was quantified using NanoDrop ND-1000. The sample preparation and microarray hybridization were performed based on the Arraystar’s standard protocols. Briefly, total RNAs were digested with Rnase R (Epicentre Technologies, USA) to remove linear RNAs and enrich circular RNAs. Then, the enriched circRNAs were amplified and transcribed into fluorescent cRNA utilizing a random priming method (Arraystar Super RNA Labeling Kit). The labeled cRNAs were hybridized onto the Arraystar Human circRNA Array V2 (8x15K, Arraystar). After washing the slides, the arrays were scanned by the Agilent Scanner G2505C. Agilent Feature Extraction software (version 11.0.1.1) was used to analyze the acquired array images. Quantile normalization and subsequent data processing was performed using the R software limma package. Differentially expressed circRNAs were identified through Fold Change filtering. Hierarchical Clustering was performed to show the distinguishable circRNAs expression pattern among samples.

### Cell lines and culture

Human normal liver cell line LO2 and HCC cell lines HepG2, 7402 and 97H were obtained from American Type Culture Collection (ATCC, USA) and passaged in our laboratory for less than 6 months after resuscitation of frozen aliquots. The cells were cultured in DMEM (Invitrogen, USA) with 10% FBS (GIBCO, Brazil) at 37 °C with 5% CO_2_. All cell lines were re-authenticated by short tandem repeat DNA profiling every 6 months after used.

### Quantitative RT-PCR analysis (qRT-PCR)

Total RNA of cells and tissues was isolated using TRIzol reagent (Life Technologies, USA). cDNA was synthesized using the PrimeScript RT reagent kit (Takara Bio Inc., China), and RT-PCR was performed using SYBR Premix Ex Taq (Takara Bio Inc.) with CFX96 Real-time PCR system (Bio-Rad, USA) to evaluate the abundance of target transcripts relative to house-keeping genes U6 or β-actin. The relative fold-change in expression with respect to a control sample was calculated by the 2-ΔΔCt method.

### CCK8 assay

Cell proliferation was assessed by CCK-8 assay (Dojindo Laboratories, Japan). Cells (1 × 10^3)^ were seeded into 96-well plates and incubated at 37 °C for 24 h before transfection. CCK-8 solution (10 μl) was added to each well 48 h after transfection. After 2 h of incubation at 37 °C, the absorbance at 490 nM was measured using Spectra Max 250 spectrophotometer (Molecular Devices, USA). Triplicate independent experiments were performed.

### Detection of cell apoptosis by JC-1 staining

To measure cell apoptosis, JC-1 staining was used (Biyuntian Biochemistry Limited Company, China). Cells were cultured in 24-well plates for 48 h post-transfection, washed with PBS and incubated with a working solution of JC-1 for 20 min at 37 °C. Then, cells were rinsed twice with PBS, stained with 1 mL of 10% DMEM medium containing 5 μmol/L JC-1. The stained cells were analyzed using a fluorescence microscope to determine color changes in the florescence from red to green.

### Matrigel invasion assay

Briefly, cells were seeded onto the EC matrix (Millipore, Germany) in the insert of 24-well culture plates. Then, 20% fetal bovine serum was added to the lower chamber as an attractant. After 48 h, the non-invading cells and the EC matrix were gently removed with a cotton swab. Invasive cells were fixed and stained with crystal violet, imaged and counted. Triplicate independent experiments were performed.

### Mouse xenograft model and in vivo metastasis assays

All animal studies were approved by the Institutional Animal Care and Use Committee (IACUC) of Xiangya Hospital, Central South University. Standard animal care and laboratory guidelines were followed according to the IACUC protocol. 4-week old BALB/c nude mice were injected subcutaneously with 2 × 10^6^ HepG2 cells (Five mice per group. A sample size of at least 4 mice per group was estimated to provied a power of 90% for a significance level of 0.05 with a two-tailed t test) and treated with intratumoral injection (40 μL si-NC or si-circFBLIM1). Tumor volumes were measured every 4 days for 28 days and calculated by the formula: volume = length × (width/2)^2^. For in vivo metastasis assays, HepG2 cells were inoculated into nude mice (five mice per group) through the tail vein. After 4 weeks, the mice were euthanized, necropsies were performed, and the lung metastatic nodules were counted. HE staining confirmed that the nodules were metastatic tumors.

### Nuclear-cytoplasmic fractionation

Cytoplasmic and nuclear RNA Isolation were performed using PARIS™ Kit (Invitrogen, USA) following the manufacturer’s instruction. Briefly, the cells were lysed with cell fractionation buffer and centrifuged to separate the nuclear and cytoplasmic cell fractions. The supernatant was transferred to a fresh RNase-free tube. The remaining lysate was washed with cell fractionation buffer and centrifuged. Cell disruption buffer was added to lyse the nuclei. Mix the lysate and the supernatant above with a 2× lysis/binding solution and add equal volume of ethanol Draw the mixture through a filter cartridge then wash the sample with wash solution. The RNA of cytoplasmic and nuclear was eluted with elution solution.

### Luciferase reporter assay

Luciferase reporter vector with the full length of the 3′-UTR of FBLIM1 or circFBLIM1 were constructed. Then we generated the mutant luciferase reporter vectors with QIAGEN XL-site directed Mutagenesis Kit (QIAGEN, California, USA). HepG2 cells were seeded into 96-well plates and co-transfected with luciferase reporter vector and miR-346 mimics or miR-346 LNA using the Lipofectamine 2000 transfection reagent. After 48 h of incubation, the firefly and Renilla luciferase activities were quantified with a dual-luciferase reporter assay (Promega, USA).

### RNA immunoprecipitation (RIP) assay

RIP assay with MS2-binding protein (MS2bp), which specifically binds RNA containing MS2-binding sequences (MS2bs) is performed to pull down endogenous miRNAs associated with circRNAs to validate the direct binding between them. Briefly, HCC cells were co-transfected with MS2bs-circFBLIM1 (construct containing circFBLIM1 transcript), MS2bs-circFBLIM1mt (the miR-346 complementary site was mutated by deletions) or control vector MS2bs-Rluc together with MS2bp-GFP using Lipofectamine 2000 (Invitrogen, USA). After 48 h, RIP was performed using GFP antibody (Roach, USA) with Magna RIP RNA-Binding Protein Immunoprecipitation Kit (Millipore, USA) according to the manufacturer’s instructions. The complexes of RNA were then treated with Trizol (Life Technologies) for further purification and the miR-346 level was analyzed by qRT-PCR.

### Western blot analysis

Western blot analysis was performed using standard procedures. Briefly, total proteins were extracted and separated by 10% SDS-PAGE and transferred onto a PVDF membrane (Millipore, USA). To block nonspecific binding, the membranes were incubated with 5% skim milk powder at room temperature for one hour. The membrane was then incubated with primary antibody against FBLIM1 (1:1000, Abcam, USA), followed by HRP-labeled secondary antibody (Santa Cruz) and detected by chemiluminescence. An anti-β-actin antibody (1:1000, Affinity, USA) was used as a protein loading control.

### Statistical analysis

Comparisons between groups were analyzed with t tests and χ^2^ tests. *P* < 0.05 was considered as statistically significant. The statistical analysis was performed using SPSS 19.0 software.

## Results

### circRNA expression profiles in HCC

**Fig. 1:**
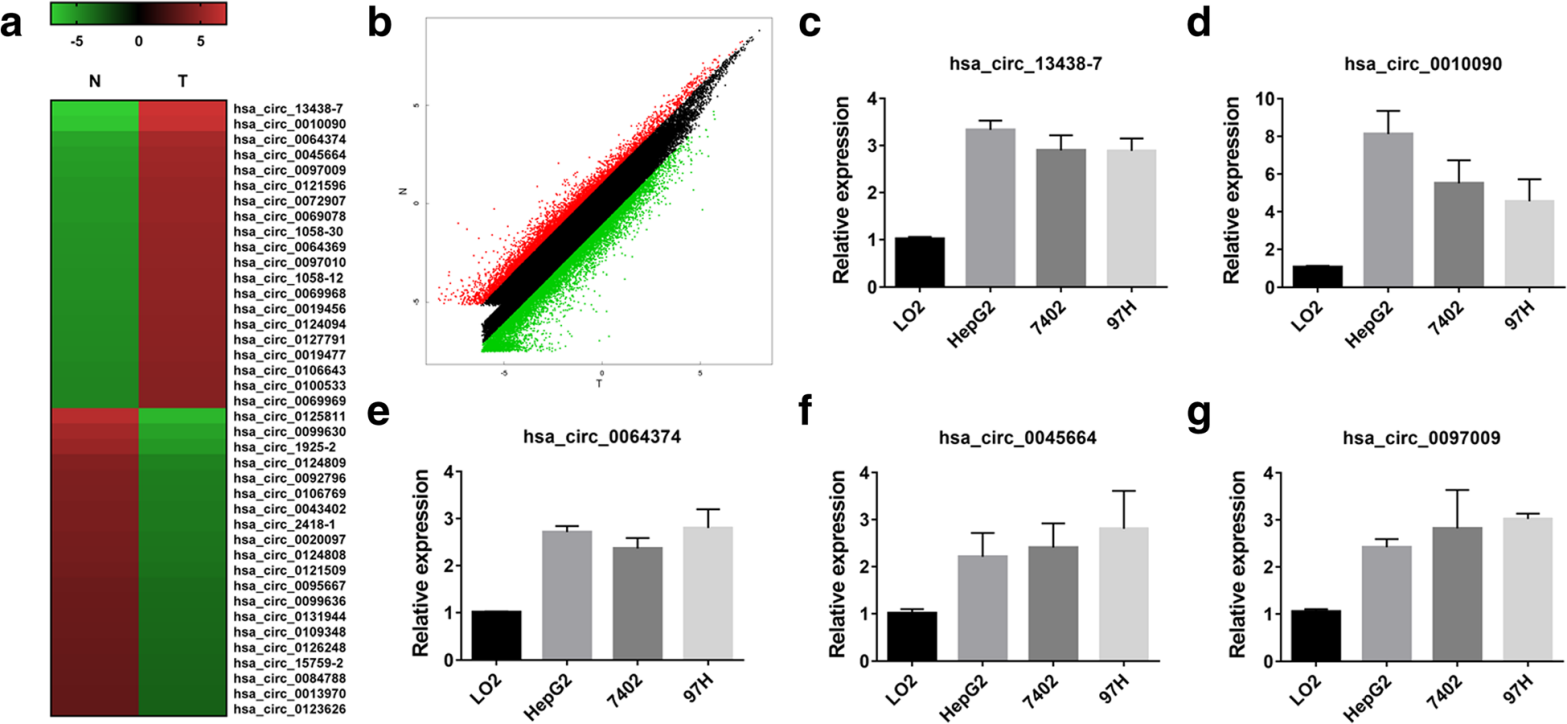
circRNA expression profiles in HCC. **a** The cluster heat map showed the top 20 upregulated and downregulated circRNAs in HCC tissues. Red color indicates high expression level and green color indicates low expression level. **b** The scatter plot revealed the variation in circRNA expression between HCC tissues and matched para-carcinoma normal tissues. The values of x and y axes were the normalized signal values of the samples (log2 scaled). **c**-**g** qRT-PCR was performed to verify the expression of the top five upregulated circRNAs in HCC cell lines. All the data are shown as the mean ± S.D

To explore the potential involvement of circRNAs in HCC, we performed circRNA microarray analyses on three pairs of HCC tissues (T) and matched para-carcinoma normal tissues (N). We found 6024 circRNAs downregulated with fold change greater than 2, *P* < 0.05 in HCC tissue, while 10,720 circRNAs upregulated by the same cutoff. Hierarchical clustering showed the top 20 upregulated and downregulated circRNAs in HCC tissues (Fig. 1a). And the variation of circRNAs expression was revealed in the scatter plot (Fig. 1b). qRT-PCR was performed to verify the expression of the top five upregulated circRNAs in three HCC cell lines (Fig. 1c-g). The hsa_circ_0010090 was the top upregulated circRNA in HCC cell lines. According to human reference genome (GRCh37/hg19), we further assumed that hsa_circ_0010090, located at chr1:16084668–16113084, is derived from gene FBLIM1 (filamin binding LIM protein 1 or FBLP1), which is located on chromosome 1p36.21. Thus, we termed hsa_circ_0010090 as “circFBLIM1”.

### Knockdown of circFBLIM1 inhibits growth and invasion, and promotes apoptosis in HCC

**Fig. 2:**
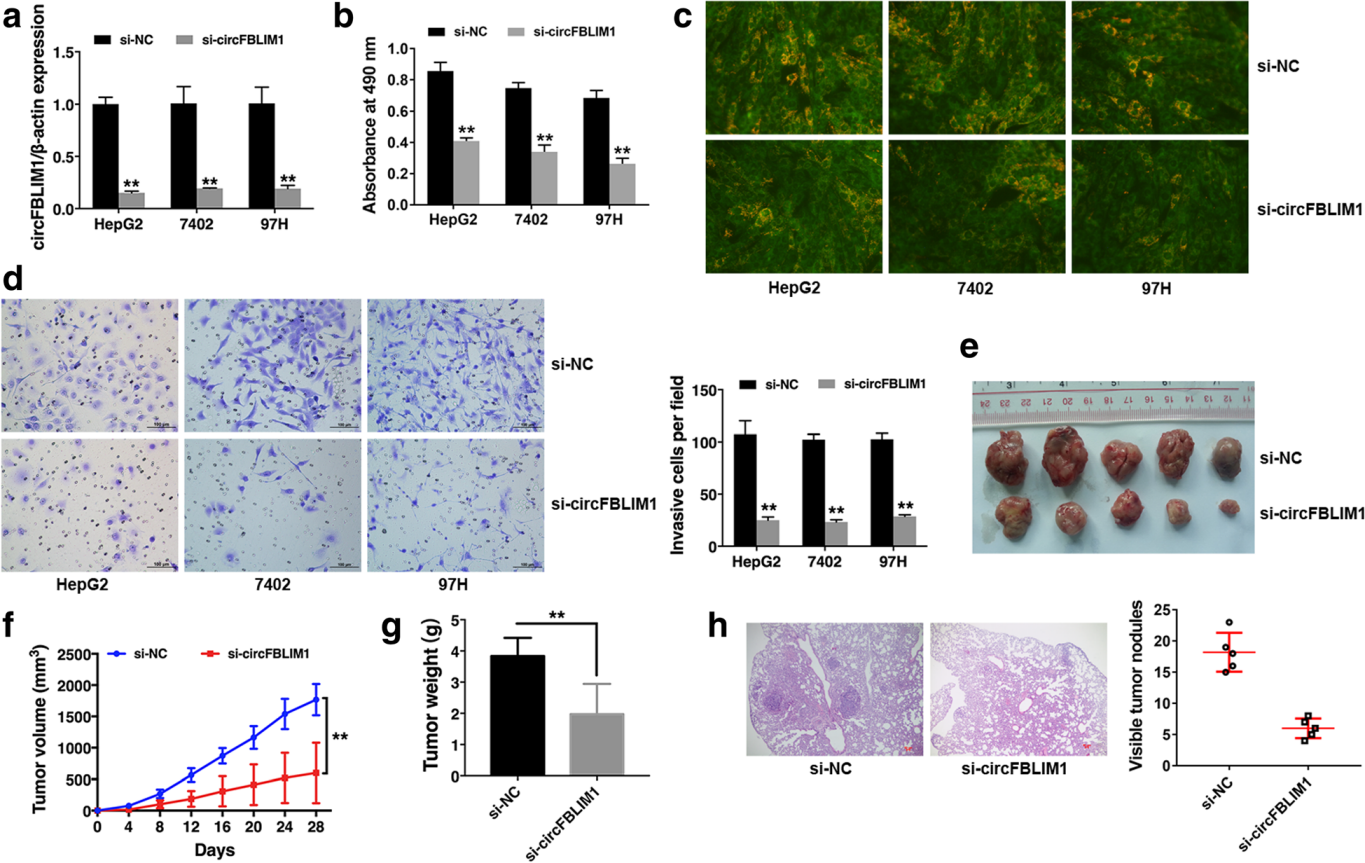
Knockdown of circFBLIM1 inhibits growth and invasion, and promotes apoptosis in HCC. **a** Cells were transfected with si-NC or si-circFBLIM1, qRT-PCR analysis demonstrated that the transfection was successful. **b** CCK8 assay was performed to assess cell growth. **c** Cell apoptosis was determined 48 h post-transfection by JC-1 staining. Green indicates apoptosis. Original magnification, × 400. **d** Matrigel invasion assay was performed. Representative images of invaded cells are shown in the left panel, and the results are summarized in the right panel. Original magnification, × 200. **e** Representative images of xenografts tumor (five mice per group) in nude mice. **f** Tumor volume was monitored every 4 days for 28 days. **g** The weights of xenograft tumors are summarized. **h** Representative images of HE stained lung metastatic nodules are shown. Original magnification, × 40. The number of metastatic nodules was quantified (five mice per group) in the right panel. All the data are shown as the mean ± S.D., **P < 0.01

Since circFBLIM1 is upregulated in HCC, we used RNA interference to knock down the expression of circFBLIM1 to evaluate its biological functions in HCC. qRT-PCR analysis demonstrated that the inhibition was successful (Fig. 2a). CCK-8 assay revealed that downregulation of circFBLIM1 significantly suppressed cell growth (Fig. 2b). Apoptosis assay showed that knockdown of circFBLIM1 resulted in increased green fluorescence in HCC cells, indicating increased cell apoptosis (Fig. 2c). Transwell assay revealed that knockdown of circFBLIM1 impaired the capacity of cell invasion (Fig. 2d). To further investigate the function of circFBLIM1 in tumor growth and invasion in vivo, xenograft experiments were performed. We found that inhibition of circFBLIM1 led to a significant decrease in tumor growth (Fig. 2e-g). And Much less visible metastatic nodules were found in si-circFBLIM1 group (Fig. 2h). All these findings suggest that circFBLIM1 knockdown suppresses cell growth and invasion, and promotes apoptosis in HCC.

### circFBLIM1 serves as a sponge for miR-346

**Fig. 3:**
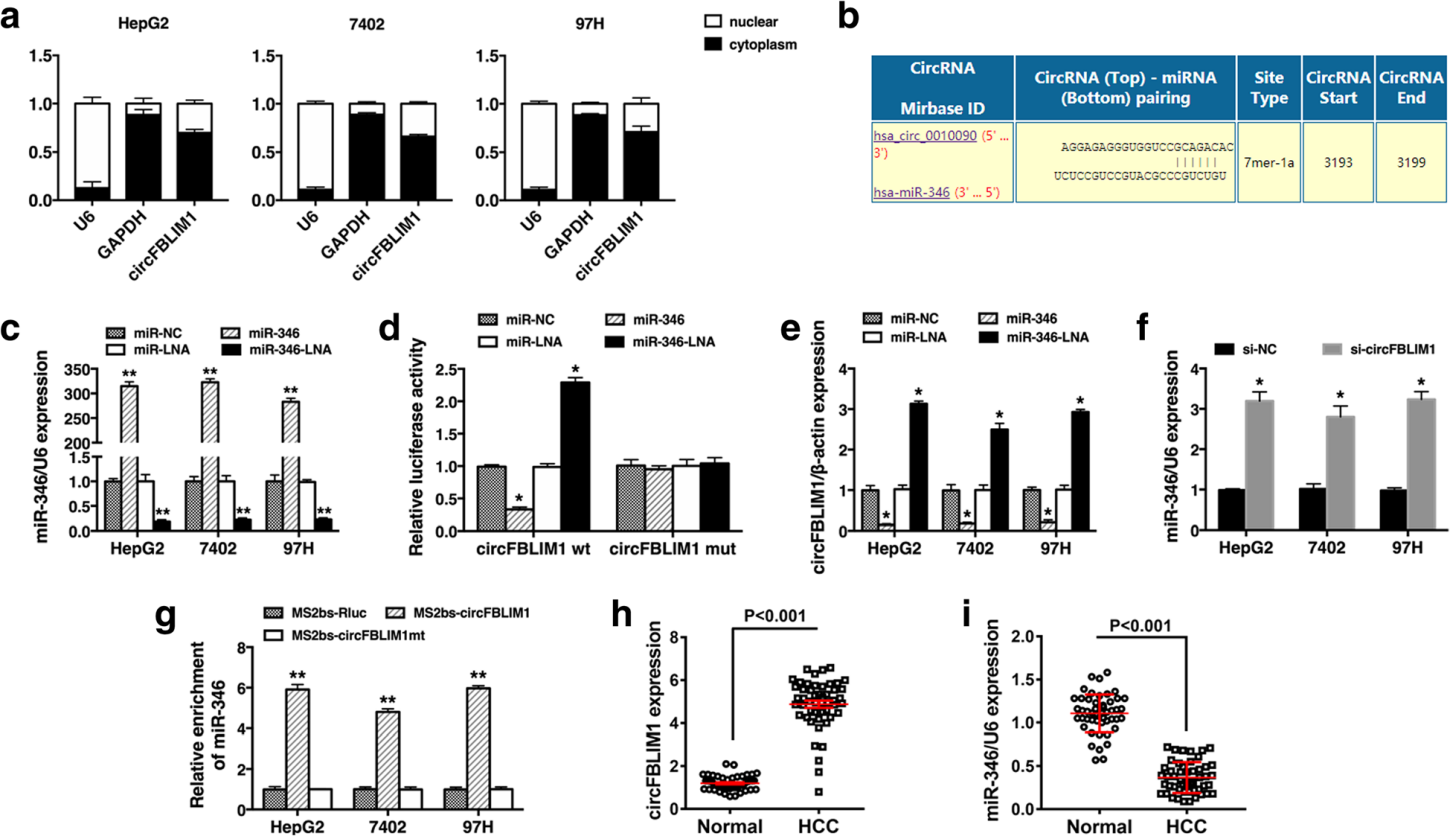
circFBLIM1 serves as a sponge for miR-346. **a** The levels of U6, GAPDH and circFBLIM1 were assessed by qRT-PCR in nuclear and cytoplasmic fractions. **b** The predicted binding sites of miR-346 within circFBLIM1 were shown. **c** Cells were transfected with miR-346 mimics or miR-346 LNA, qRT-PCR analysis demonstrated that the transfection was successful. **d** Luciferase assay of cells co-transfected with miR-346 mimics or miR-346 LNA and luciferase reporter containing circFBLIM1 (circFBLIM1 wt) or mutant construct (circFBLIM1 mut). **e** Cells were transfected as described, and the expression of circFBLIM1 was determined by qRT-PCR. **f** Cells were transfected with si-NC or si-circFBLIM1, and the expression of miR-346 was determined by qRT-PCR. U6 snRNA was used as an internal control. **g** MS2bp-MS2bs based RIP assay in HCC cells transfected with MS2bs-circFBLIM1, MS2bs-circFBLIM1mt, or MS2bs-Rluc. **h** The expression level of circFBLIM1 in 50 HCC tissues and matched para-carcinoma normal tissues was determined by qRT-PCR. **i** The expression level of miR-346 in the aboved tissues was determined by qRT-PCR. All the data are shown as the mean ± S.D., *P < 0.05 and **P < 0.01

Next, we characterized the intracellular location of circFBLIM1 in HCC cell lines. Nuclear and cytoplasmic fractions were separated from cells and the levels of the nuclear control U6 and cytoplasmic control GAPDH were detected by qRT-PCR, respectively. The results revealed that circFBLIM1 mostly distributed in the cytoplasm of HCC cells (Fig. 3a). As circFBLIM1 was predominantly localized in the cytoplasm, it might function as a ceRNA to sequester miRNAs, leading to the liberation of corresponding miRNA-targeted transcripts. We explored whether circFBLIM1 could act as a miRNA sponge and potential binding sites of miR-346 were found within the circFBLIM1 sequence (Fig. 3b). Thus, we performed luciferase reporter assays to determine whether miR-346 could bind to circFBLIM1. qRT-PCR analysis demonstrated that the transfection was successful (Fig. 3c). The luciferase intensity was reduced by more than 40% when cells were co-transfection with luciferase reporters and miR-346 mimics, and increased when cells were co-transfection with luciferase reporters and miR-346 LNA (Fig. 3d). However, co-transfection of mutated luciferase reporter and miR-346 mimics or miR-346 LNA had no significant effect on luciferase activity (Fig. 3d). qRT-PCR analysis further confirmed that miR-346 mimics could inhibit the expression of circFBLIM1 while miR-346 LNA increased circFBLIM1 expression (Fig. 3e). And the expression of miR-346 was upregulated after circFBLIM1 inhibition (Fig. 3f).

To further validate the direct binding between circFBLIM1 and miR-346, we performed MS2bp-MS2bs based RIP assay. Figure 3g showed that miR-346 was significantly enriched in RNAs retrieved from MS2bs-circFBLIM1 compared with that from MS2bs-circFBLIM1mt or control MS2bs-Rluc, indicating the specific interaction between circFBLIM1 and miR-346. Moreover, the expression of circFBLIM1 was upregulated in HCC tissues, while miR-346 was downregulated (Fig. 3h-i). In line with luciferase assays, our data confirmed that circFBLIM1 functionally interacts with miR-346 and serves as a sponge for miR-346 in HCC.

### circFBLIM1 and FBLIM1 act as ceRNAs in HCC through regulation of miR-346

**Fig. 4:**
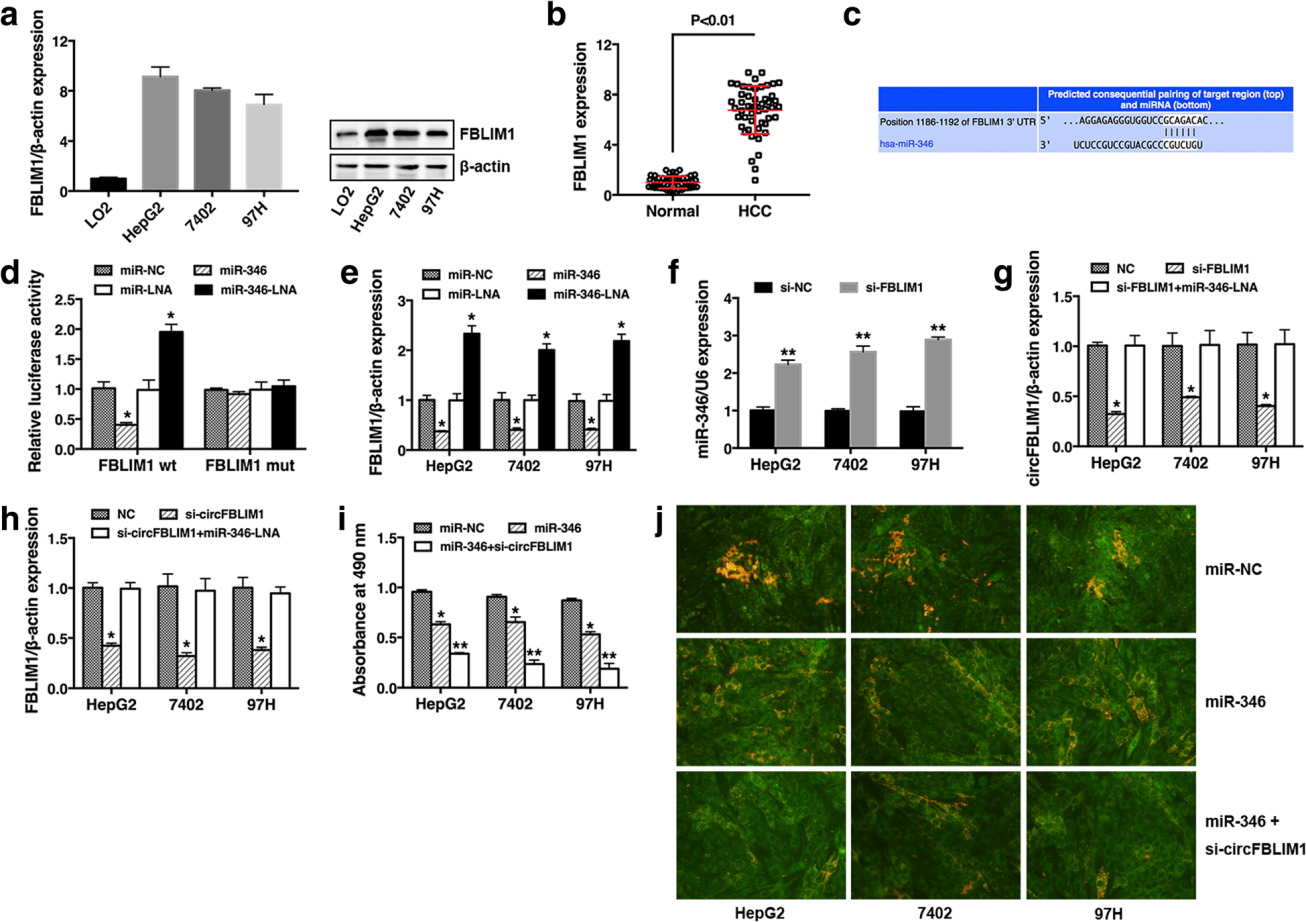
circFBLIM1 and FBLIM1 act as ceRNAs in HCC through regulation of miR-346. **a** The expression level of FBLIM1 was determined by qRT-PCR (left) and Western blot (right) in HCC cell lines. β-actin was used as a control. **b** The expression level of FBLIM1 in 50 HCC tissues and matched para-carcinoma normal tissues was determined by qRT-PCR. **c** The predicted binding sites of miR-346 within FBLIM1 were shown. **d** Luciferase assay of cells co-transfected with miR-346 mimics or miR-346 LNA and luciferase reporter containing FBLIM1 3′-UTR (FBLIM1 wt) or mutant construct (FBLIM1 mut). **e** Cells were transfected as described, and the expression of FBLIM1 was determined by qRT-PCR. **f** Cells were transfected with si-NC or si-FBLIM1, and the expression of miR-346 was determined by qRT-PCR. **g** Cells were transfected with NC, si-FBLIM1 or si-FBLIM1 + miR-346 LNA, and the expression of circFBLIM1 was determined by qRT-PCR. **h** Cells were transfected with NC, si-circFBLIM1 or si-circFBLIM1 + miR-346 LNA, and the expression of FBLIM1 was determined by qRT-PCR. **i** Cells were transfected with miR-NC, miR-346 or miR-346 + si-circFBLIM1, and CCK8 assay was performed. **j** Cells were transfected as described, and apoptosis assay was performed. Original magnification, × 400. All the data are shown as the mean ± S.D., *P < 0.05 and **P < 0.01

To explore whether circFBLIM1 acts as a ceRNA to sequester miR-346 and liberate the expression of FBLIM1, we continued to detect the expression of FBLIM1 in HCC cell lines and tissues. The results showed that FBLIM1 was also upregulated in HCC cell lines (Fig. 4a) and tissues (Fig. 4b). Furthermore, we used TargetScan to identify the putative target genes of miR-346 and FBLIM1 was predicted (Fig. 4c). To confirm this finding, luciferase reporter assay was performed. A significant decrease of luciferase activity was observed when co-transfected with miR-346 mimics, but not with mutant luciferase reporter (Fig. 4d). On the contrary, luciferase activity significantly increased when co-transfected with miR-346 LNA (Fig. 4d). qRT-PCR validated that the expression of FBLIM1 was reduced by miR-346 and increased with miR-346 LNA (Fig. 4e). Moreover, the expression of miR-346 was upregulated after FBLIM1 inhibition (Fig. 4f). These results indicated that FBLIM1 was a direct target of miR-346 and could also sequester miR-346.

Next, we detected the expression of circFBLIM1 after knockdown of FBLIM1 and found a repression of circFBLIM1 (Fig. 4g). To prove whether FBLIM1 functions as a ceRNA, we co-transfected miR-346 LNA and si-FBLIM1 and found that the repression was reversed (Fig. 4g). We further detected the expression of FBLIM1 after circFBLIM1 inhibition and observed a decreased FBLIM1 expression. And it was also reversed when co-transfection with miR-346 LNA (Fig. 4h). These results suggest that circFBLIM1 and FBLIM1 serve as ceRNAs by harboring miR-346.

Since circFBLIM1 could serve as a ceRNA to regulate the progress of HCC by sequestering miR-346, we further explored whether over expression of miR-346 and knock down of circFBLIM1 could inhibit HCC progress synergistically. Subsequent cell proliferation assay and apoptosis assay revealed that miR-346 combined with circFBLIM1 inhibition further suppressed cell growth and promoted cell apoptosis in HCC (Fig. 4i-j).

## Discussion

Although surgical technique and radio-chemotherapy regimens have been improving in the past decades, the survival rate of HCC has not increased as we expect. Therefore, it is urgent to explore the molecular mechanisms of carcinogenesis and progression in HCC to develop novel strategies to improve HCC prognosis [3, 17].

circRNAs are covalently closed, single-stranded transcripts and identified as a naturally occurring family of noncoding RNAs [18]. Many miRNAs and lncRNAs have been reported to regulate HCC development. However, there are currently few reports describing the role of circRNAs in HCC. In this study, many aberrantly expressed circRNAs in HCC were identified. We found that circFBLIM1 was upregulated in HCC tissues and cell lines. Further experiments showed that knockdown of circFBLIM1 inhibited cell growth and invasion, and promotes apoptosis in HCC. These results revealed that circFBLIM1 plays a vital role in HCC progression and may be a biomarker and therapeutic target for HCC.

To date, more and more miRNAs have been found to take vital parts in the pathogenesis and progression of cancers. miR-346 is reported to regulate multiple cellular processes, including carcinogenesis, inflammatory response and differentiation [19–23]. Guo J et al. found that miR-346 enhances the expression of AGO2 to regulate the activity of other miRNAs and the migration and invasion of cervical cancer [24]. Xiao J et al. demenstrated that miR-346 overexpression could suppress high glucose-induced EMT in mouse podocytes [25]. Due to the significant functions of miR-346 in cancer, development of miR-346-based gene therapy is encouraged for multiple types of cancers.

FBLIM1, also known as Migfilin, is a novel LIM domain-containing protein present both at cell-matrix [26] and cell–cell adhesions [27]. Recently, a few studies have implicated FBLIM1 in cancer metastasis [28–31]. FBLIM1 is crucial for cell migration in a variety of cell types and its depletion impairs cell migration [32]. Expression of FBLIM1 correlates with tumor grade and poor prognosis and promotes migration and invasion in glioma [33]. Gkretsi V et al. found that FBLIM1 is associated with a more aggressive HCC phenotype, that FBLIM1 could be a potential therapeutic target against HCC metastasis [34].

Recently, circRNAs have been shown to act as miRNA sponges to regulate gene expression [15, 35, 36]. Zheng Q et al. reported that circHIPK3 directly binds to miR-124 and inhibits miR-124 activity to function as a cell growth modulator [37]. Han D et al. found that circRNA MTO1 acts as the sponge of miR-9 to suppress HCC progression [8]. And circRNA_100338 functions as an endogenous sponge for miR-141-3p to regulate HCC invasion. In this study, we found that miR-346 could target both circFBLIM1 and FBLIM1, suggesting that circFBLIM1 might function as miR-346 sponge to regulate FBLIM1 expression through ceRNA mechanism. There are several lines of evidence implicating that circFBLIM1 functions as a ceRNA to FBLIM1 in HCC as a sponge of miR-346. First, bioinformatics analyses showed that the 3′UTR of FBLIM1 and circFBLIM1 contain binding sites for miR-346. Second, luciferase reporter assays and MS2bp-MS2bs based RIP assay verified this prediction. Third, knockdown of circFBLIM1 reduced FBLIM1 expression. Finally, inhibition of miR-346 reversed the effect of circFBLIM1 knockdown. All the above results suggest that circFBLIM1 and FBLIM1 is a couple of ceRNAs that are linked by miR-346.

## Conclusions

Taken together, our study indicates that circFBLIM1 is upregulated in HCC. Knockdown of circFBLIM1 inhibits growth and invasion, and promotes apoptosis in HCC. And circFBLIM1 functions as a ceRNA to regulate FBLIM1 expression by decoying miR-346. circFBLIM1 can be used as a diagnostic biomarker and potential target in HCC therapy.

## Abbreviations

ceRNA: Competing endogenous RNA
circFBLIM1: Circular RNA FBLIM1
circRNAs: Circular RNAs
HCC: Hepatocellular cancer
MS2bp: MS2-binding protein
MS2bs: MS2-binding sequences
qRT-PCR: Quantitative real-time PCR
RIP: RNA immunoprecipitation
